# Modelled Cost-Effectiveness of a Package Size Cap and a Kilojoule Reduction Intervention to Reduce Energy Intake from Sugar-Sweetened Beverages in Australia

**DOI:** 10.3390/nu9090983

**Published:** 2017-09-06

**Authors:** Michelle Crino, Ana Maria Mantilla Herrera, Jaithri Ananthapavan, Jason H. Y. Wu, Bruce Neal, Yong Yi Lee, Miaobing Zheng, Anita Lal, Gary Sacks

**Affiliations:** 1 The George Institute for Global Health, University of New South Wales, Sydney 2042, Australia; jwu1@georgeinstitute.org.au (J.H.Y.W.); bneal@georgeinstitute.org.au (B.N.); 2 School of Public Health, Faculty of Medicine, The University of Sydney, Sydney 2042, Australia; 3 School of Public Health, Faculty of Medicine, University of Queensland, Brisbane 4001, Australia; ana.mantillaherrera@uqconnect.edu.au (A.M.M.H.); y.lee5@uq.edu.au (Y.Y.L.); 4 Deakin Health Economics, Centre for Population Health Research, Deakin University, Geelong 3220, Australia; jaithri.ananthapavan@deakin.edu.au (J.A.); anita.lal@deakin.edu.au (A.L.); 5 The Royal Prince Alfred Hospital, Sydney 2042, Australia; 6 School of Public Health, Faculty of Medicine, Imperial College, London SW7 2AZ, UK; 7 Queensland Centre for Mental Health Research (QCMHR), The Park Centre for Mental Health, Wacol 4076, Australia; 8 Global Obesity Centre, Centre for Population Health Research, Deakin University, Geelong 3220, Australia; j.zheng@deakin.edu.au (M.Z.); gary.sacks@deakin.edu.au (G.S.)

**Keywords:** obesity prevention, cost-effectiveness, portion size, sugar-sweetened beverages, economic evaluation

## Abstract

Interventions targeting portion size and energy density of food and beverage products have been identified as a promising approach for obesity prevention. This study modelled the potential cost-effectiveness of: a package size cap on single-serve sugar sweetened beverages (SSBs) >375 mL (*package size cap*), and product reformulation to reduce energy content of packaged SSBs (*energy reduction*). The cost-effectiveness of each intervention was modelled for the 2010 Australia population using a multi-state life table Markov model with a lifetime time horizon. Long-term health outcomes were modelled from calculated changes in body mass index to their impact on Health-Adjusted Life Years (HALYs). Intervention costs were estimated from a limited societal perspective. Cost and health outcomes were discounted at 3%. Total intervention costs estimated in AUD 2010 were AUD 210 million. Both interventions resulted in reduced mean body weight (*package size cap*: 0.12 kg; *energy reduction*: 0.23 kg); and HALYs gained (*package size cap*: 73,883; energy *reduction*: 144,621). Cost offsets were estimated at AUD 750.8 million (*package size cap*) and AUD 1.4 billion (*energy reduction*). Cost-effectiveness analyses showed that both interventions were “dominant”, and likely to result in long term cost savings and health benefits. A package size cap and kJ reduction of SSBs are likely to offer excellent “value for money” as obesity prevention measures in Australia.

## 1. Introduction

Over the past 30–40 years, the portion-size of many packaged food and beverage products has increased significantly [1,2,3,4,5,6]. Package and portion size are known to influence the quantity of food an individual selects and consumes [5,6,7]. When offered larger packages or portions of food or beverages, individuals are known to consume more and are unlikely to compensate by increasing their physical activity or reducing the quantity of other foods and beverages eaten at the same sitting or later in the day [6,8,9,10,11,12]. The sustained provision of large portion sizes of nutrient poor but energy dense foods and beverages may be an important contributor to obesity and non-communicable diseases (NCD) [8,11].

Initiatives targeting portion and package size (henceforth referred to as portion size) have been identified as a promising approach to reduce obesity and obesity-related NCDs [13,14,15]. To-date, the majority of initiatives targeting portion size have been voluntary, with uptake at the discretion of the food industry [15]. These interventions have targeted portion size in two main ways: (i) through a reduction in the quantity of the product provided, predominantly through a change in package size; or (ii) by reducing the energy density per serve of the food or beverage product (reformulation) [15]. The proposed New York City (NYC) ban of very large servings of sugar-sweetened beverages (SSBs) is the most high profile example of the first approach, although its implementation was unsuccessful [16]. The pledge by sugary drink companies to reduce the sugar content and energy density of their beverages as a part of the UK’s Public Health Responsibility Deal [17], is an example of the second approach.

In 2011–2012, in Australia, 63% of adults and 25% of children were classified as overweight or obese, with overweight and obesity deemed the second highest contributor to the burden of disease in Australia [18]. Similar to other countries, many Australians currently consume poor quality diets with nearly a third of energy coming from discretionary foods [19]. The latest dietary survey results showed that 34% of Australians consume SSBs, which contributed to 4% of total energy consumed and 17% of total sugars consumed for individuals aged two years and older [19]. Furthermore, the survey highlighted that consumption of SSBs was higher for children aged 2–18 years than adults (47% and 31%, respectively) [19]. The Australian government has identified tackling obesity as a priority action [20,21], and changes in portion size as a key target [21]. Despite strong interest by government and the food industry, the potential effectiveness and cost-effectiveness of implementing such strategies to reduce obesity at a population level has not been investigated. We therefore estimated the potential cost-effectiveness of implementing: (i) a package size cap on single-serve products (*package size cap*); and (ii) product reformulation to lower energy density (*energy reduction*) on packaged SSBs available for sale in Australia.

## 2. Materials and Methods

### 2.1. Overview

This evaluation is one of several that have been undertaken using standardised methods as part of an obesity prevention priority setting study in Australia. A proportional multi-state, multiple cohort life table model (Obesity model) was used to estimate lifetime health benefits (Health-Adjusted Life Years (HALYs)) and costs of changes in body mass index (BMI) arising from changes in kilojoule (kJ) consumption due to each intervention. Interventions were assumed to operate at their full effectiveness potential and were compared against a “no intervention” scenario where the distribution of BMI in the 2010 Australian population remains unchanged. We present the effectiveness and cost-effectiveness of a series of scenarios, in which key parameters influencing the intervention effect were varied to test the validity of assumptions.

### 2.2. Specification of the Interventions

#### 2.2.1. A Package Size Cap on all Packaged Single-Serve SSBs (*Package Size Cap*)

The first intervention modelled was specified as a *package size cap* of 375 mL on packaged single-serve SSBs sold in Australia. This applied to all pre-packaged single-serve SSBs, but excluded SSBs produced on-site, such as post-mix typically sold in restaurants. A product was deemed a single-serve product if: (i) the package size was equal to the serving size, as per the nutrition information panel of the product; or (ii) the servings per pack was indicated as “1” on the product label. These single-serve or individual portion pack products are commonly consumed in one sitting [22]. The cap size was selected based on recommendations in the Australian Dietary Guidelines (ADG) which specify that a serving of discretionary food, such as SSB, should provide about 600 kJ which translates to approximately 375 mL (1 can) [23].

#### 2.2.2. Reformulation of SSBs to a Reduced Energy Density (*Energy Reduction*)

This intervention was specified as the reformulation of all SSBs available for sale in Australia to a reduced energy density. A 5% kJ reduction and 30% kJ reduction were modelled as this aligned with current reformulation examples in the United Kingdom under the Public Health Responsibility Deal Calorie Reduction Pledge [17]. We did not specify the nutrient composition changes that would be required to achieve this reduction in energy density; however, it is likely that sugar would be reduced, as sugar is the primary contributor to the energy content of SSBs. It has also been documented by food manufacturers that have pledged to the UK’s Public Health Responsibility Deal Calorie Reduction Pledge that they have reduced sugar in SSBs in order to reduce overall energy content [17,24]. We did not specify a particular package size or focus on single-serve beverages, as it was unlikely that manufacturers would reformulate for one pack size only.

### 2.3. Estimation of Effect Size

**Figure 1:**
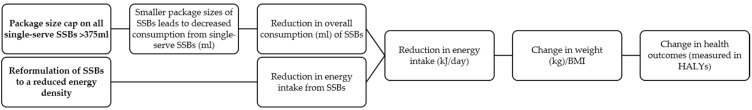
Logic pathway for modelling the effect of the SSB package size cap and reformulation of SSBs to a reduced energy density interventions for obesity prevention. HALYs: health-adjusted life years; BMI: body mass index; SSBs: sugar-sweetened beverages.

Past randomised controlled trials conducted in experimental settings have demonstrated that changing portion size is an effective strategy to reduce weight gain and obesity; however, to our knowledge there is no robust evidence regarding the effectiveness of policies targeting portion size interventions directed as SSB at the population level. Therefore, a range of different scenarios and associated assumptions were used to determine the likely range of possible effects for each intervention, using a logic pathway of the likely intervention effect (Figure 1) and based on the best available evidence. A summary of these scenarios can be found in Table 1 and the assumptions and rationale for these scenarios can be found in Table 2. For both interventions, the latest available food consumption data for the Australian population from the 2011–2012 Australian Health Survey (AHS) [25] were used as a starting point to model how each intervention would alter mean daily energy intake (by age and sex groups) through a change in consumption. Energy intake changes were assumed to be sustained over the lifetime of the population since both interventions target a macro-environmental change to the food system [26,27,28,29].

### 2.4. Package Size Cap Intervention

Evidence suggests that the package size of a product influences the total quantity of food or beverage an individual consumes, especially in the case of single-serve or individual portion packaging [6,7]. Experimental studies indicate that providing smaller portion or package sizes results in individuals consuming less food and beverages overall; however, these studies were in highly controlled experimental settings [6,8,32]. In the absence of effectiveness data on the proposed portion size cap implemented at a country level, various theoretical scenarios were developed and evaluated.

Mean daily consumption (mL/day and kJ/day) of SSBs was derived from the 2011–2012 AHS [25] for all ages and sex groups, based on the following food categories: sugar-sweetened carbonated beverages, sugar-sweetened flavoured waters, sugar sweetened flavoured iced teas, sugar-sweetened sports/electrolyte drinks and sugar-sweetened cordials. Fruit drinks and fruit juices were not included as we were unable to distinguish consumption of fruit juice with no added sugars (considered as “core” food by the ADGs) to fruit drinks and fruit juice with added sugars. Consumption data in relation to specific package sizes were not available from the AHS. Therefore, we estimated the total consumption of single-serve SSBs and single-serve SSBs >375 mL using customer purchase data from a recent Australian Food Labelling Trial (FLT) [37,38]. These data included self-recorded purchases (from till receipts) of each category of SSBs from a non-representative sample (*n* = 1578) of Australians, over a period of 4 weeks [37,38]. Consumption (purchase) data were only available at the household level, not the individual level. Consequently, we used volume (mL) of SSBs sold to determine the proportion of SSBs that are single-serve. Product data were sourced from the Australian FoodSwitch (FS) Database (further details: Dunford et al. 2014 [39]), which provided product name, category, pack(age) size, nutrition and serving size information. We matched products from the FLT dataset to the FS dataset using information from the product barcode.

The combined FLT and FS dataset was used to calculate the proportion of units and volume (mL) of single-serve and non-single-serve SSBs sold by package size. For all package sizes of single-serve SSBs > 375 mL, we calculated the volume greater than 375 mL. We then multiplied this volume by the proportion of consumption related to each package size. The proportional reductions were summed and then applied uniformly across the AHS consumption data to determine the overall reduction in SSB consumption and, hence, the corresponding mean daily kJ reductions, by age and sex groups.

The base case scenario assumed no compensatory drinking in relation to the reduced portion size. Additional analyses examined the following scenarios: (i) some compensatory drinking (25% of consumers continue to drink >375 mL of SSBs in different formats such as 3 × 200 mL package sizes); and (ii) 10% of consumers substitute their usual SSB to the same package size of a sugar-free (0 kJ) alternative.

Scenarios were modelled under two modes of implementation of the intervention: (i) mandatory implementation of the intervention, whereby the Australian government imposed a legislative ban, and 100% of manufacturers adhered to the *package size cap*; and (ii) voluntary implementation, whereby manufacturers would pledge to adhere to the *package size cap* (see Table 1 for details of the different scenarios examined). Based upon initial data from the implementation of another voluntary initiative in Australia, the Health Star Rating front of pack labelling system, we assumed that only 20% of eligible products would adopt the voluntary *package size cap* [30,40].

### 2.5. Energy Reduction Intervention

The effect size for this intervention was derived by applying a percentage reduction to the energy intake related to SSBs, from AHS data for each age and sex group. The intervention effect was calculated as the difference between mean daily energy intake before and after the intervention was applied. Unlike the *package size cap*, the intervention effect applied to all SSBs, and was not limited to single-serve products or those of a particular package size.

We modelled different levels of kJ reductions (5% and 30%) and degrees of implementation (mandatory: all SSBs reformulated; voluntary: 20% of SSB reformulated, Table 1). It was assumed that there was no compensatory drinking for SSBs that were reformulated, as supported by prior experimental studies that consumers continue to consume the same quantity of foodstuff without compensating for changes in kJ [41].

### 2.6. Intervention Costs

Intervention costs were assessed from a limited societal perspective. Costs directly related to the intervention included: costs to the government, non-government organisations (NGOs), and costs to the food industry (see Table 3). Specific costs to government included the costs of passing legislation in the Australian Parliament (for relevant scenarios) [36]. Other cost considerations for governments included estimates for promoting, educating, monitoring and overseeing the implementation and upkeep of the interventions, which was consistently applied to all scenarios implemented on a mandatory basis (refer to Table 1: *package size cap* intervention, scenarios A1–A3; *energy reduction* intervention, scenarios B1 and B2). Costs to NGOs covered advocacy, marketing and promotion of the interventions and was applied to all scenarios.

Costs to the food industry were derived based on previous analyses of expected costs of implementation and maintenance of a public health-related intervention (Health Star Rating; HSR) affecting packaged food in Australia, including costs for industry, government and non-government organisations [30]. Estimates provided in this report leverage previous data prepared for Food Standards Australian New Zealand (FSANZ) [42] which estimated the per unit price when undertaking changes to food and beverage labelling through literature reviews and input from FSANZ and industry stakeholders. Detailed estimates for costs to industry include costs for proofing, packaging re-design (changes to package size and shape), labelling changes and labour, ingredients, overhead and implementation costs (technical, scientific, executive and administrative) [30,42]. It is acknowledged that costs to the food industry for the different interventions will differ, however it was assumed the same based on the best evidence currently available. For voluntary implemented scenarios (refer to Table 1: *package size cap* intervention, scenarios A4–A6; *energy reduction* intervention, scenarios B3 and B4), costs were applied at a reduced rate (20% of costs instead of 100%), reflecting the lower level of expected implementation.

## 3. Modelling Cost-Effectiveness

Changes in energy intake (kJ) at the population level (by age and sex) were converted to changes in body weight (kg) using validated energy balance equations for children (aged 2–19) and adults (aged ≥ 20) [43,44]. These changes in weight were converted to changes in BMI using average Australian height and weight by sex, for single-year age groups (children) and five-year age groups (adults) from the AHS 2011 [25].

The altered distribution of BMI as a result of the interventions was applied to the Obesity model to estimate lifetime HALYs. The Obesity model quantifies changes in the total mortality and morbidity of the 2010 Australian population resulting from changes in the epidemiology of obesity-related diseases (i.e., incidence, prevalence and mortality) and the independent impact of non-disease obesity on quality of life. Nine causally obesity-related diseases were included: ischaemic heart disease, hypertensive heart disease, ischemic stroke, diabetes, colorectal cancer, kidney cancer, breast cancer, endometrial cancer and osteoarthritis [45]. Total cost offsets were the result of health care cost savings attributable to the intervention as a result of reduced incidence of obesity related diseases. Total HALYs and costs were estimated comparing: (i) a reference population that represents the current BMI distribution and disease patterns of a cohort of the 2010 Australian population; and (ii) an intervention population that is identical to the reference population but includes the impact of the intervention on the BMI distribution of the population. The modelling was conducted for the Australian population aged 2–100 years, over their lifetime.

All costs and benefits were discounted at 3% and are expressed in 2010 values. The Health Price Index from the Australian Institute for Health and Welfare (AIHW) [46] was used to adjust costs to 2010 AUD as required. The Incremental Cost Effectiveness Ratio (ICER) was calculated by dividing the incremental net costs by incremental health benefits of the intervention compared to current practice. Further details on the model can be found elsewhere [47].

### Uncertainty Analysis

The estimates for each cost element and the changes in weight, BMI and HALYs resulting from the interventions were estimated as means with 95% uncertainty intervals (UIs). Monte Carlo simulation (2000 iterations) was used to estimate parameter uncertainty using Ersatz (version 1.35) software—an Excel add-in [48]. ICERs are presented on a cost-effectiveness plane, which demonstrates the range of plausible ICERs for each intervention and associated scenarios. Interventions that are both cost saving and increase health benefits are considered “dominant”. If the intervention is more costly and more effective than current practice, a willingness to pay threshold of AUD 50,000 per HALY gained [49] is used to determine cost-effectiveness.

## 4. Results

### 4.1. Changes in Consumption, Energy Intake and Body Weight

Estimates of the likely impact of the *package size cap* on single-serve beverages >375 mL on changes in consumption and the resultant changes in energy intakes are presented in Table 4. Of the SSBs currently sold in Australia, we estimated that 27% of all SSBs sold were single-serve. The intervention would affect 16% of SSBs overall, with approximately two thirds of single-serve SSBs (59%) being greater than 375 mL. The effect of the package size intervention differed across the population based on current consumption of SSBs. The base case scenario would result in an average change in consumption of SSBs from 564.4 kJ/day to 550 kJ/day across the entire population. This mean decrease in energy intake of 14.4 kJ/day translates in to a 0.12 kg weight reduction and 0.05 BMI unit reduction. Effect estimates were observed to have a larger impact on males across all age groups. Furthermore, teenagers aged 13–19 years (both sex) and males aged ≥20 years were also observed to benefit most from the intervention, with weight reductions of 0.15 kg and 0.17 kg, respectively. Details of the estimate of effects by age and sex can be seen in Table 4.

Reformulation of all SSBs to a reduced energy density would affect 100% of SSBs sold in Australia (*n* = 3226 stock keeping units; SKUs), and 71 known brands. The base case 5% kJ reduction of SSBs resulted in a higher average change in consumption of SSBs than the *package size cap* intervention: from 564.4 kJ/day to 536.8 kJ/day, across the entire population. This also attributed to higher weight and BMI reductions across the population (0.23 kg, 0.10 BMI units; Table 4). As with the *package size cap*, teenagers and males aged ≥20 years were seen to benefit most from the intervention. For both interventions, estimated effects were observed to be substantially higher for scenarios that were implemented on a mandatory basis (scenarios A1–A3; B1–B2; Supplementary Table S1). As expected, the intervention involving a 30% kJ reduction (scenario B2) had the greatest effect on change in energy consumption, weight and BMI, followed by scenario A3 and A6. The scenarios observed to have the least estimated effect were those implemented on a voluntary basis (scenarios A5, A6 and B3).

### 4.2. Costs

Input cost parameters for each intervention are presented in Table 3. Estimated intervention costs to achieve the modelled change in kJ consumption through a portion size cap or a kJ reduction intervention vary between scenarios, but fall within a range of AUD 44.6 M (95% UI: 31.6 M–57.1 M) to AUD 210.5 M (95% UI: 147.5 M–270.3 M). Government-imposed legislative scenarios for both the *package size cap* and reformulation interventions had a higher intervention cost (AUD 209.7 M; 95% UI: 147.7 M–272.9 M) than the scenario modelling a voluntary change by manufacturers (AUD 44.5 M; 95% UI: 31.4 M–57.5 M) (Table 5). However, mandatory regulation also resulted in greater health outcomes (total HALYs gained range: 55,581 to 822,835) compared to scenarios modelling voluntary implementation (total HALYs gained range: 11,043 to 289,045). The bulk of the costs of both interventions fall on industry (Table 3).

### 4.3. Cost-Effectiveness Results

**Figure 2:**
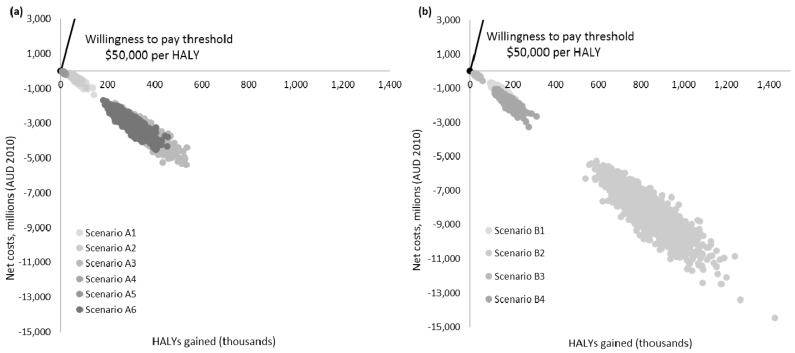
Cost-effectiveness planes of the (**a**) package size cap and (**b**) energy reduction interventions and associated scenarios.

Our results indicate that both interventions, in all respective scenarios, were dominant—i.e., they were both cost saving and resulted in additional HALYs gained (Table 3 and Figure 2). Over the lifetime of the 2010 Australian population, the seemingly modest changes in weight as a result of the base case *package size cap* intervention translated to substantial health gains and total net cost savings (Table 3). This result was consistent across all scenarios for this intervention, with scenarios implemented under a mandatory assumption (scenarios A1–A3) resulting in better cost-effectiveness results than those modelled under a voluntary assumption (scenarios A4–A6). Scenarios with the SSB substitution (scenarios A3 and A6) were estimated to have substantial increases in benefits than other *package size cap* scenarios modelled.

For the base case kJ reduction intervention, the changes in weight across the population (all age and sex groups) translated to a substantive change in HALYs gained and total net costs (Table 5). This was also observed for all scenarios modelled in this intervention. As expected, scenarios modelled under the assumption of a 30% kJ reduction (scenarios B2 and B4) demonstrated a five-fold increase in benefits compared to scenarios modelled under a 5% kJ reduction (scenarios B1 and B3). When compared to the *package size cap*, the kJ reduction intervention was observed to have larger benefits.

For both interventions, scenarios modelled based on a voluntary implementation (A3–A6, and B3–B4) resulted in lower implementation costs. However, these interventions were also much less cost-effective due to lower HALYs gained and lower cost savings. Both interventions were estimated to have beneficial impacts on mortality rates across seven of the nine modelled obesity-related diseases. The kJ reduction intervention was observed to have almost double the benefits with an average 26,420 lives saved in comparison to the 13,590 lives saved as a result of the *package size cap*. For both interventions, the larger changes in mortality were projected for ischemic heart disease, followed by diabetes, kidney cancer and stroke.

## 5. Discussion

This cost-effectiveness analysis showed that both the *package size cap* and *energy reduction* interventions were likely to be “dominant” (both effective and cost saving) in the Australian context under current modelling assumptions (Figure 2). This modelling exercise suggests that policy-based population wide interventions such as these are likely to offer excellent “value for money” as obesity prevention measures, especially if implemented on a mandatory basis.

Results from the modelled scenarios confirm the current consensus that population-level portion size interventions are a promising approach to addressing obesity and obesity-related NCDs [13,14,15] and therefore should be considered by policy makers. There is a lack of cost-effectiveness studies that investigate the impact of population-based interventions that change the food environment. Such studies are needed for policy makers to make informed decisions on how to spend limited resources [50]. To provide further context to policy makers as to whether these proposed interventions are a worthwhile investment, the results of these analyses should be interpreted in relation to other comparable cost-effectiveness studies in the Australian setting. To-date, only one other study has used a similar standardised methodology to evaluate the cost-effectiveness of active transport on obesity-related health outcomes [47]. This study found that active transportation interventions result in fewer health benefits relative to their cost. It follows that interventions targeting positive change in the food environment may be more impactful than those seeking to increase levels of active transportation. Another cost-effectiveness study from the broader ACE-Prevention study [51], evaluated the implementation of traffic light labelling (TLL) and a junk food tax targeting at Australian adults [29]. This study and other interventions from ACE-Prevention which sought to implement changes to the food environment were found to be dominant compared to “program-based” food interventions [51]. The results of our study were comparable to a more recent study which modelling the impact of a tax on SSBs on the 2010 Australian adult population [52].

The primary strength of this analysis is the policy relevance of the interventions chosen. In Australia in 2015, the Healthy Food Partnership (the Partnership) was established as the primary government-led initiative to address food reformulation in relation to NCD prevention. The Partnership consists of the Australian Government, food industry bodies and leading public health groups that have agreed to work cooperatively to tackle obesity, encourage health eating and empower food manufacturers to make positive changes to their products [21]. The interventions modelled in this research strongly align to two of the three core objectives of the Partnership (portion size and reformulation) [53]. Using multiple scenarios and varying realistic assumptions, this research has provided the opportunity to assess the potential impacts of such public health initiatives on the Australian population.

The limitations of this study centre around the quality of evidence relating to intervention effectiveness. Direct evidence supporting the likely impact of the interventions on consumer behaviour is weak. To counter this uncertainty, we made conservative assumptions to estimates of change in energy consumption; in addition to modelling multiple scenarios of intervention effectiveness. Another limitation involved the estimation of consumption of single-serve SSBs. As data on SSB consumption by package size were not available from nationally representative dietary surveys, we had to use a calculated estimate using sales data from a relatively small intervention trial. This may have underestimated the actual consumption of single-serve SSBs. As more evidence of these effects of these types of interventions becomes available, these assumptions can be revisited.

Furthermore, as definitive data on the costs for changes in packaging and reformulation could not be sourced, estimated costs to industry are not well substantiated. However, for the *package size cap* intervention, as food manufacturers are reducing their products to a package size that already exists (375 mL), we think the estimates are likely to be conservative. Additionally, although it is acknowledged that implementing a *package size cap* or *energy reduction* may be more complex than estimates of implementing changes to front of pack labelling (from which the cost estimates were derived), we believe that if the food industry were given sufficient lead time, the costs to changing packaging and reformulation would be significantly reduced because of changes in product packaging and formulation that occur as a part of natural product-lifecycles. Our model did not take in to account the potential loss of revenue to the food industry and its subsequence impact on consumers. It is likely that these costs would be recovered from re-distribution of sales (e.g., sales lost from 600 mL SSBs would be replaced from sales from 375 mL), however it is also possible that some costs may be passed on to the consumer, especially for the small-to-medium sized manufacturers. Additionally, as with all population-based models, our results represent a simplified version of reality. However, input parameters included 95% uncertainty intervals determined by the Monte Carlo simulation (2000 iterations) using Ersatz (version 1.35).

The implications of this study are that both a *package size cap* and *energy reduction* on SSBs are likely to be highly cost-effective and have sizeable effects on population health. Despite the degree of uncertainty around the size of benefits, both interventions should be considered for implementation in Australia. Interventions chosen to be modelled were based on a global review of current literature and policies [15], and reflected currently implemented Calorie Reduction Pledges as proposed in the United Kingdom’s Responsibility Deal [17]. Furthermore, our chosen interventions also reflected portion size related public health initiatives in the United States, such as the proposed New York City ban on 16oz SSBs [54] and the Health Weight Commitment pledge. Comparisons of government implemented legislative scenarios and voluntary scenarios highlighted the importance of food industry adherence if these interventions were implemented in a real-world setting. Both HALYs gained and healthcare system savings were higher in the government legislated scenarios, than the voluntary scenarios (Supplementary Table S1). The role of government support is thus of great importance to the success of these interventions [55].

Further research in this area could be undertaken to investigate other scenarios for the design and implementation on both interventions modelled in this analyses. For example, this model could be used to analyse the effects of a *package size cap* or *energy reduction* targeted at different food categories such as confectionery, ready meals (including those sold at Quick Service Restaurants), snack foods and foods specifically designed for school canteens. Furthermore, this model could be used to look at the potential impact of pricing food items proportionate to package size. Many countries are taking action around SSBs, including SSB taxes, and it would also be valuable to model the impact of combined interventions targeting SSBs. Importantly, it would also be imperative to perform research into consumer acceptability of portion size interventions, particularly because consumer acceptance studies have indicated that such initiatives may be perceived by consumers to restrict their freedom of choice [32,56].

## 6. Conclusions

This study provides an overview of the potential health benefits and cost-effectiveness of two potential population-based, SSBs portion size interventions in Australia. Our estimates demonstrate that both a *package size cap* and *energy reduction* in SSBs are likely to have significant health-related benefits and the potential for healthcare system cost savings. Estimates further highlight the critical role of food industry adherence to achieve the best outcomes from the interventions, and strongly support the role of government to implement and oversee such initiatives. In the Australian context, the current Healthy Food Partnership provides a potential vehicle for the government to demonstrate such leadership in this area.

## Figures and Tables

**Table 1:** Scenarios modelled.

**Intervention: Package Size Cap on Single-Serve Sugar Sweetened Beverages (SSBs)**	
*Scenario A1 (base case)*	Government imposes legislation banning the sale of single-serve, packaged SSBs greater than 375 mL. No compensatory eating
*Scenario A2*	Government imposes legislation banning the sale of single-serve, packaged SSBs greater than 375 mL. 25% compensatory eating, for example, 25% of individuals continue to consume the same volume of SSB but in different formats (e.g., 3 × 200 mL)
*Scenario A3*	Government imposes legislation banning the sale of single-serve, packaged SSBs greater than 375 mL. 10% of individuals substitute SSBs for equivalent single-serve portions (>375 mL) of sugar-free alternatives
*Scenario A4*	Voluntary industry pledge to cease production of single-serve, packaged SSBs greater than 375 mL. No compensatory eating
*Scenario A5*	Voluntary industry pledge to cease production of single-serve, packaged SSBs greater than 375 mL. 25% compensatory eating, for example, 25% of individuals continue to consume the same volume of SSB but in different formats (e.g., 3 × 200 mL)
*Scenario A6*	Voluntary industry pledge to cease production of single-serve, packaged SSBs greater than 375 mL. 10% of individuals substitute SSBs for equivalent single-serve portions (>375 mL) of sugar-free alternatives
**Intervention: Energy Reduction (Reformulation) of SSBs**	
*Scenario B1 (base case)*	Government imposes legislation to reduce kJ/serve by 5% for all SSBs. No compensatory consumption
*Scenario B2*	Government imposes legislation to reduce kJ/serve by 30% for all SSBs. No compensatory consumption
*Scenario B3*	Voluntary industry pledge to reduce kJ/serve by 5% for all SSBs. No compensatory consumption
*Scenario B4*	Voluntary industry pledge to reduce kJ/serve by 30% for all SSBs. No compensatory consumption

**Table 2:** Parameters, assumptions and rationale for modelled scenarios.

Parameter	Assumption	Rationale	Source
Government-implemented interventions	Government legislation and 100% adherence by food industry	The cost of legislation has been incorporated. Given that monitoring of non-compliance is relatively simple, it is assumed that there is 100% compliance by the food industry.	
Voluntary interventions	Assumed 20% adherence by food industry	Based on the Health Star Rating System Cost Benefit Analysis report	[30]
		Latest estimates indicate 14.4% uptake rate of the voluntary Health Star Rating system in Australia	[31]
Consumption patterns	All age groups consume single-serve SSB unit sizes in the same proportion	Insufficient data to calculate differences in age and sex groups.	
Compensatory eating ^a^—package size cap	Assumed 25% of individuals would still consume the same portion sizes (>375 mL) irrespective of the portion size cap Assumed 10% of individuals would swap to sugar-free alternatives in order to continue to consume the same portion sizes (>375 mL)	Consumer dietary recalls indicate that 27.3% of participants ate an additional snack outside of the workplace cafeteria where there was controlled portion restrictions	[32]
		United States based modelling of the New York City ban on SSBs would affect 80% of consumer consumption behaviour	[33]
		The 2011–2012 Australian Health Survey found that approximately 10% of individuals drink sugar-free (made with intense sweetener) beverages	[19]
No compensatory eating ^a^—package size cap	Assumed that individuals that usually would consume >375 mL would move on to the next largest available portion size	Based on estimates in other modelling studies and interventions in controlled experimental settings	[32,33]
	It is also assumed that individuals are unlikely to pay for multiple, smaller (<375 mL) single serve pack sizes of SSBs to compensate for their past consumption behaviour of >375 mL of SSBs	Single-serve portion sizes are typically consumed in the one setting	[34]
No compensatory eating ^a^—kilojoule reduction	Assumed individuals would not purchase multiple or increased volume of SSBs to compensate for kJ reduction	Research has indicated that it is unlikely people would consume more as the total volume remains the same	[35]
Costs—passing legislation	Assumed this cost would only occur once, in the first year of the intervention		[36]
Costs—industry and NGO (marketing and promotion)	It is assumed these costs would only occur in the first 2 years during the “implementation phase” of the intervention	Once industry and NGO have completed the implementation of the new portion size, there is no further costs attributable to the intervention	[30]
Costs—government (promotion, education, enforcement and oversight/monitoring)	It is assumed that these costs will occur for the first 5 years of the intervention	Based on the Health Star Rating System Cost Benefit Analysis report	[30]
Kilojoule reduction	Assumed to be applied to all SSBs, not specific portion-sizes	If the food industry reformulated, they would reformulate the recipe for all portion sizes, it would be too costly and inconvenient to reformulate for a specific portion size only	
Kilojoule reduction—5% and 30% reduction targets	Assumed that these are reasonable and achievable targets for food industry to meet	Reductions in 5% and 30% of energy density across SSB have been self-reported by food manufacturers as a part of the Public Health Responsibility Deal’s Calorie Reduction Pledge	[17]
	It is assumed that reduction in sugar content will be how food industry would meet this target		
Sugar-free SSB alternatives	Assumed to have 0 kJ	No other macronutrients are present in SSBs that would contribute to energy density (kJ content)	

^a^ Compensatory eating refers to compensatory drinking for the purposes of this paper. SSB: sugar-sweetened beverage; NGO: non-government organization.

**Table 3:** Intervention costs (adjusted to 2010 AUD) with associated uncertainty distributions and assumptions.

Cost Description	Intended Payer of Cost	Values (AUD Million)	Distribution ^c^	Sources and Assumptions
Cost of implementing new legislation ^a^	Government	1.0 (95% CI: 0.9–1.2)	Gamma	Most likely value based upon estimates by [36]. Assumed that this cost would only occur once.
Costs of administering, enforcing, promoting, educating, monitoring and overseeing the implementation of either the package size cap or energy reduction interventions ^b^	Government	12.3 (range: ±50%)	Pert	Estimate based on projected cost of implementing “Health Star Rating” front of pack labelling in Australia [30,42].
Costs of labelling and packaging changes (design, materials, proofing), labour, ingredients, overhead and implementation costs (technical, scientific, executive, administrative) ^b^	Food industry	36.9 (range: ±50%)	Pert	Estimate based on projected cost of implementing “Health Star Rating” front of pack labelling in Australia [30,42].
Costs of advocating, marketing and promoting either the package size cap or energy reduction interventions ^b^	Non-government organisations	5.5 (range: ±50%)	Pert	Estimate based on projected cost of implementing “Health Star Rating” front of pack labelling in Australia [30,42].

All amounts are in AUD million, with 2010 as the reference year. ^a^ Only used for scenarios involving mandatory implementation. ^b^ Cost estimates were based on 20% adherence by manufacturers were multiplied by 5 to obtain 100% for scenarios involving mandatory implementation. For scenarios involving voluntary implementation, 20% of the cost values presented in the table were used. ^c^ Due to a lack of data on the cost of implementing these interventions, wide uncertainty intervals have been used in the modelling.

**Table 4:** Estimated effects of package size cap (base case) and energy reduction (base case) interventions on the 2010 Australian population over their lifetime.

		Average Energy Intake (Baseline) (kJ/Day/person)	Average Consumption from SSBs before Intervention (kJ/Day/person)	Average Consumption from SSBs after Intervention (kJ/Day/Person)		Estimated Change in Energy in Response to Intervention (kJ/Day/Person)		Average Body Weight (kg) (Baseline)	Average Change in Weight in Response to Intervention (kg)		Average Change in BMI in Response to Intervention (kg/m^2^)	
				Package Size Intervention (Base Case)	Energy Reduction Intervention (Base Case)	Package Size Intervention (Base Case)	Energy Reduction Intervention (Base Case)		Package Size Intervention (Base Case)	Energy Reduction Intervention (Base Case)	Package Size Intervention (Base Case)	Energy Reduction Intervention (Base Case)
Aged 2–12	Male	8140.3	466.6	454.7	443.2	−11.9	−23.3	38.5	−0.06	−0.12	−0.04	−0.07
	Female	7137.4	426.9	416.1	405.6	−10.9	−21.4	38.4	−0.06	−0.12	−0.04	−0.08
Aged 13–19	Male	10,771.7	687.0	669.5	659.0	−17.5	−29.0	90.4	−0.15	−0.24	−0.05	−0.08
	Female	8260.6	600.8	585.5	570.7	−15.3	−30.0	77.6	−0.15	−0.29	−0.05	−0.11
Aged ≥ 20	Male	10,308.0	684.8	667.3	650.5	−17.5	−34.4	103.1	−0.17	−0.34	−0.06	−0.11
	Female	7841.2	557.6	543.4	529.7	−14.2	−27.9	78.4	−0.14	−0.28	−0.05	−0.11
Total population		8664.8	564.4	550.0	536.8	−14.4	−27.6	71.1	−0.12	−0.23	−0.05	−0.10

**Table 5:** Cost-effectiveness analyses for the package size cap and energy reduction interventions ^a^.

	Package Size Cap Intervention						Energy Reduction Intervention			
	Scenario A1 (Base Case)	Scenario A2	Scenario A3	Scenario A4	Scenario A5	Scenario A6	Scenario B1 (Base Case)	Scenario B2	Scenario B3	Scenario B4
Average HALYs gained (95% UI)	73,883 (57,038; 96,264)	55,581 (42,240; 72,671)	348,236 (267,567; 455,788)	14,781 (11,260; 19,170)	11,043 (8389; 14,670)	289,045 (220,900; 379,533)	144,621 (109,050; 189,848)	822,835 (641,097; 1,050,183)	28,981 (21,884; 37,976)	173,410 (131,057; 226,732)
Total intervention costs (AUD; 95% UI)	209.7 M (147.7; 272.9)	209.7 M (147.7; 272.9)	209.7 M (147.7; 272.9)	44.5 M (31.4; 57.5)	44.5 M (31.4; 57.5)	44.5 M (31.4; 57.5)	209.7 M (147.7; 272.9)	209.7 M (147.7; 272.9)	44.5 M (31.4; 57.5)	44.5 M (31.4; 57.5)
Total cost-offsets (AUD; 95% UI) ^b^	−750.9 M (−991.4; −555.7)	−556.6 M (−762.3; −422.1)	−3.5B (−4.8; −2.6)	−150.5 M (−201.3; −111.9)	−112.9 M (−151.2; −84.3)	−2.9B (−3.9; −2.2)	−1.5 B (−1.9; −1.1)	−8.3 B (−10.8; −6.4)	−295.0 M (−390.8; −217.3)	−1.8 B (−2.4; −1.3)
Net costs (AUD; 95% UI) ^b^	−540.9 M (−792.5; −340.9)	−356.9 M (−564.2; −194.8)	−3.3B (−4.5; −2.4)	−106.1 M (−159.8; −66.0)	−68.4 M (−108.3; −36.2)	−2.8B (−3.8; −2.2)	−1.3 B (−1.7 B; −868.8 M)	−8.1 B (−10.6; −6.2)	−250.6 M (−346.8; −217.3)	−1.7 B (−2.3; 1.3)

M: million; B: billion; HALYs: health adjusted life years; UI: uncertainty intervals. ^a^ The upper and lower limit of 95% UI for all scenarios were dominant: cost saving and improved health outcomes. ^b^ Negative costs represent cost savings.

## Supplementary Materials

The following are available online at www.mdpi.com/2072-6643/9/9/983/s1, Table S1: Estimated effects of package size cap and energy reduction interventions on the 2010 Australian population over their lifetime.
