# Supplementary material for: Modelled Cost-Effectiveness of a Package Size Cap and a Kilojoule Reduction Intervention to Reduce Energy Intake from Sugar-Sweetened Beverages in Australia
Source: Nutrients. 2017 Sep 6;9(9):983. doi: 10.3390/nu9090983 (PMC5622743; doi:10.3390/nu9090983)
Supplement: Supplementary file 1 [file nutrients-09-00983-s001.zip › nutrients-214932-supplementary.pdf]

Online supplementary materials

Supplementary table S1**: *Estimated effects of package size cap and energy reduction interventions on the 2010 Australian population over their lifetime****

Modelled scenarios		Average energy intake (baseline) (kJ/day/person)	Average consumption from SSBs before intervention (kJ/day/person)	Average consumption from SSBs after intervention (kJ/day/person)	Estimated change in energy in response to intervention (kJ/day/person)	Average body weight (baseline) (kg)	Average change in weight in response to intervention (kg)	Average change in BMI in response to intervention (kg/m^2)
**Package size cap intervention**	**Scenario A1 (base case)**	8,664.8	564.4	550.0	-14.4	71.1	-0.12	-0.05
	**Scenario A2**	8,664.8	564.4	553.6	-10.8	71.1	-0.09	-0.04
	**Scenario A3**	8,664.8	564.4	497.0	-67.4	71.1	-0.57	-0.23
	**Scenario A4**	8,664.8	564.4	561.5	-2.88	71.1	-0.02	-0.01
	**Scenario A5**	8,664.8	564.4	562.3	-2.16	71.1	-0.02	-0.01
	**Scenario A6**	8,664.8	564.4	508.5	-55.9	71.1	-0.47	-0.19
**Energy reduction intervention**	**Scenario B1 (base case)**	8,664.8	564.4	536.8	-27.6	71.1	-0.23	-0.10
	**Scenario B2**	8,664.8	564.4	398.7	-165.7	71.1	-1.39	-0.56
	**Scenario B3**	8,664.8	564.4	558.8	-5.6	71.1	-0.05	-0.02
	**Scenario B4**	8,664.8	564.4	530.5	-33.9	71.1	-0.29	-0.11

*data presented is for the entire population (all ages and sexes)
